# Next Generation Sequencing of Pooled Samples: Guideline for Variants’ Filtering

**DOI:** 10.1038/srep33735

**Published:** 2016-09-27

**Authors:** Santosh Anand, Eleonora Mangano, Nadia Barizzone, Roberta Bordoni, Melissa Sorosina, Ferdinando Clarelli, Lucia Corrado, Filippo Martinelli Boneschi, Sandra D’Alfonso, Gianluca De Bellis

**Affiliations:** 1 Institute for Biomedical Technologies, National Research Council, Segrate (MI), Italy; 2 Department of Science and Technology, University of Sannio, Benevento, Italy; 3 Interdisciplinary Research Center of Autoimmune Diseases IRCAD, University of Eastern Piedmont, Novara, Italy; 4 Department of Health Sciences, University of Eastern Piedmont, Novara, Italy; 5 Laboratory of Human Genetics of Neurological Disorders, Institute of Experimental Neurology (INSPE), Division of Neuroscience, San Raffaele Scientific Institute, Milan, Italy; 6 Department of Neurology, Division of Neuroscience, Scientific Institute San Raffaele, Milan, Italy

## Abstract

Sequencing large number of individuals, which is often needed for population genetics studies, is still economically challenging despite falling costs of Next Generation Sequencing (NGS). *Pool-seq* is an alternative cost- and time-effective option in which DNA from several individuals is pooled for sequencing. However, pooling of DNA creates new problems and challenges for accurate variant call and allele frequency (AF) estimation. In particular, sequencing errors confound with the alleles present at low frequency in the pools possibly giving rise to false positive variants. We sequenced 996 individuals in 83 pools (12 individuals/pool) in a targeted re-sequencing experiment. We show that Pool-seq AFs are robust and reliable by comparing them with public variant databases and *in-house* SNP-genotyping data of individual subjects of pools. Furthermore, we propose a simple filtering guideline for the removal of spurious variants based on the Kolmogorov-Smirnov statistical test. We experimentally validated our filters by comparing Pool-seq to individual sequencing data showing that the filters remove most of the false variants while retaining majority of true variants. The proposed guideline is fairly generic in nature and could be easily applied in other Pool-seq experiments.

Population genetics studies and epidemiological studies on the genetics of multifactorial diseases require sequencing a large number of genomes at high coverage. This is mandatory both in order to reach sufficient power for case-control analysis and to compare the patterns of genetic variations across populations. Despite substantial reduction in the cost of NGS in recent years, sequencing a large number of individual genomes at high coverage is still economically challenging. An alternative cost-effective approach is to sequence DNA from pools of individuals (*Pool-seq*), which has other benefits like needing less DNA from each single individual and reducing overall work and time of sequencing experiments. Pooling allows even small labs to carry out population genetics studies, which are otherwise impossible due to exorbitant costs. However, pooling of DNA creates new problems and complexity in data analysis. One of the most challenging problems of Pool-seq is to correctly identify rare variants (allele frequency, AF < 0.01), as sequencing errors confound with the alleles present at low frequencies in the pools. Rare variants are not only abundant in population but also have potential functional roles^1^,^2^. Hundreds of Genome Wide Association Studies (GWAS) targeting common variants explain only a fraction of genetic heritability in complex diseases^3^. This implies that we need to look beyond “common disease/common variant (CD/CV)” hypothesis and genetic burden of many rare variants of small effect size with high penetrance might play key roles in explaining missing heritability of complex diseases^4^,^5^. Thus accurate determination of rare variants is extremely important in genetic disease research.

One of the key interests of population genetics study is the information about polymorphic sites and corresponding AF of variant alleles in the population. The power of many genetic analyses depends upon accurate determination of AFs of variants. In principle, Pool-seq should give more robust estimate of AF due to the larger sample size, which allows decreasing the overall *variance* of the estimated AF^6^. This hypothesis is well supported by mathematical models under the assumption that there are no sequencing errors and each individual contributes equal amount of DNA to the pools^7^,^8^,^9^. However, in reality the sequencing errors are appreciable^10^,^11^ and achieving equimolar concentration of each individual’s DNA in the pools is also somewhat difficult, which makes it worthwhile to verify the accuracy of AFs in Pool-seq experiments.

In the present study, involving targeted re-sequencing of 996 individuals in 83 pools, we show that Pool-seq can be used to accurately estimate AFs of variant alleles. By comparing Pool-seq with several public variant databases and SNP-array data of individuals constituting the pools, we show that the Pool-seq AFs are robust and reliable. We also provide general filtering guideline in order to remove spurious variants due to sequencing errors. We *individually* sequenced and identified variants for all subjects of a single pool and compared them with the results of Pool-seq, showing that the proposed filters provide a low rate of false positive and false negative variants, thus proving the utility and efficacy of the filters.

## Results and Discussion

### Sequencing results

We sequenced 84 pools (12 individuals per pool) on Illumina GaIIx sequencer after multiplexing six pools per lane following targeted capture of the genomic regions of interest (totaling 1.9 Mb). After demultiplexing, reads from each pool were tested for quality in terms of duplicate level and mapping ability in the target regions. One of the pools did not pass the Quality Control (QC), thus it was re-sequenced without success and was consequently discarded from any further analysis.

The overall sequencing performance is shown in Supplementary Table S1. We generated 13.96 millions reads per pool on average. After duplicate removal (average 5.97%), we got an average of 13.71 millions reads mapped on the human genome. On average, the mean depth was 351.9× with more than 85% of the targeted regions covered by NGS reads in each pool. Supplementary Fig. S1 shows the distribution of coverage for all pools. On average, 75% (range 70 to 80%) of the target regions are covered at least 50× and 69% are covered at least 100×. In order to quickly appreciate the sequencing quality issues concerning samples, we plotted the number of total and mapped reads (Supplementary Fig. S2) as well as mean depth and coverage (Supplementary Fig. S3) for all pools. As a general comment, a fairly homogeneous behavior in terms of sequencing was found.

### Variant Call

We have used the Pool-seq variant caller CRISP^12^ [**C**omprehensive **R**ead analysis for **I**dentification of **S**ingle Nucleotide Polymorphisms (SNPs) from **P**ooled sequencing] for the identification of the variants. CRISP is able to identify both rare as well as common variants from pooled NGS data. It has shown reasonably low false positive and false negative rates of variants on real data sets^12^,^13^. CRISP applies a sophisticated set of techniques to distinguish between false variants coming from sequencing errors compared to those from real variant alleles. In particular, CRISP analyzes the entire set of reads across all pools that cover any particular variant position, and gathers various signals in multiple steps to distinguish sequencing errors from real variants^12^.

**Figure 1:**
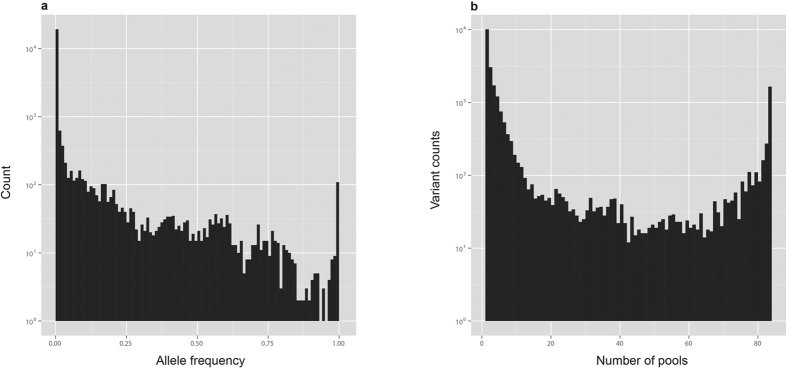
(**a**) Allele Frequency distribution of all variants. **(b)** Distribution of variants according to the number of pools in which they are found.

CRISP called a total of 29736 variants in our data out of which 27529 were single nucleotide variants (SNVs) and 2207 were insertions and deletions (INDELs). INDELs represent a challenging issue for any variant calling software and we decided to focus our attention only on SNVs. Only 23651 SNVs passed all filtering imposed by CRISP (e.g. low-depth, strand-bias etc.). Figure 1(a) shows the allele frequency (AF) distribution of all SNVs. Most variants (N = 19139, 80.92%) can be classified as rare, showing AF below 0.01. Many of the SNVs (N = 10111, 42.75%) are found in only one pool, and they may be private rare variants (present in only one individual of that pool) [Fig. 1(b)]. These are expected results since rare variants are abundant in population^1^,^2^ and their chances of detection increase with increasing sequencing depth and number of individuals sequenced. However, they could also derive from sequencing errors.

### Public database annotations

9204 out of 23651 SNVs were found in 1000Genomes database^14^ considering all populations of 1000Genomes (1000Genomes_ALL), of which 7068 were found considering the European population (1000Genomes_EUR) only. 10280 variants were found in dbSNP^15^, 1669 in ExAC^16^ (Exome Aggregation Consortium) database and 1111 in ESP^17^ (Exome Sequencing Project) database. Overall, we found 12991 (54.93%) “novel” variants not found in any of the 1000genomes, dbSNP, ExAC or ESP database. Almost all of them (n = 12780, 98.38%) are rare variants (AF < 0.01) (Supplementary Fig. S4).

### Comparison with 1000genomes and ExAC

Estimation of population AF is susceptible to sampling errors, especially if the number of samples is low. The advantage of pooling is that the variance due to sampling error can be greatly reduced by choosing a fairly large pool size. There are reports showing that the accuracy of AF-estimation in Pool-seq is comparable to, if not better than, that of individual sequencing^13^,^18^,^19^. To ascertain the accuracy of AF in our experiment, we compared it with public databases and with AF got from individual genotyping using SNP-array, as explained in the following sections.

**Figure 2:**
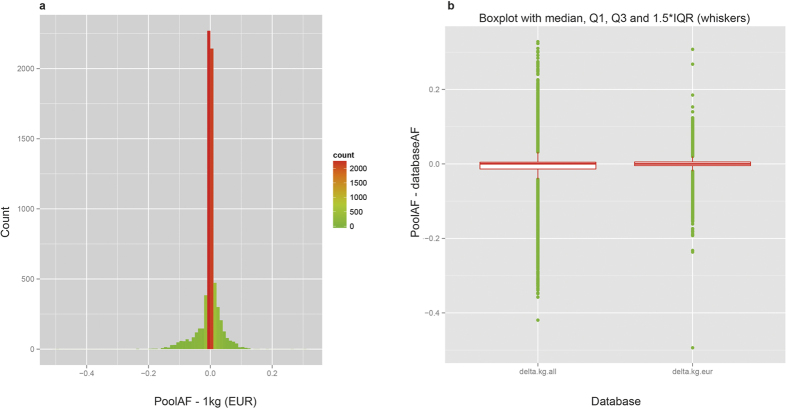
Comparison of poolAF with AF of 1000genomes. **(a)** Histogram of differences between poolAF and 1000genomes European population [1 kg(EUR)]. Minimum: −0.494;1^st^ Quartile: 0.005; Median: 0.000; Mean: −0.002; 3^rd^ Quartile: 0.005; Maximum: 0.308. **(b)** Boxplot of differences: Left panel 1000genomes_ALL (delta.kg.all) and Right panel 1000genomes_EUR (delta.kg.eur). The overall similarity between poolAF and 1000Genomes is higher for 1000genomes_EUR population as shown by smaller IQR and lesser spread of data.

The number of individuals in our samples (12 individuals/pool * 83 pools = 996 individuals) is comparable to that of 1000genomes database. We compared Pool-seq AF (poolAF) with AF of 1000Genomes_EUR population. For 7068 SNVs for which 1000genomes_EUR frequency was available, there is an excellent correlation between poolAF and 1000genomes_EUR AF (R^2^ = 0.980; Supplementary Fig. S5). The difference between poolAF and 1000genome_EUR AF shows a very tight distribution centred at zero [median = 0; Inter Quartile Range, IQR = 0.01; Fig. 2(a)]. Considering the fact that our pools are composed of Italian subjects, the overall similarity between poolAF and 1000Genomes AF is higher for 1000genomes_EUR population than 1000genomes_ALL population as expected (R^2^_EUR = 0.980 vs. R^2^_ALL = 0.922; Supplementary Fig. S5). This is also proved by the fact that the distribution of differences between poolAF and 1000Genomes AF shows smaller IQR and a much lesser spread of data for comparison with 1000genomes_EUR population than 1000genomes_ALL population [Fig. 2(b)]. In a stratified analysis for rare and common variants separately, we further show that the relative differences (absolute delta/AF) are small for either of the groups of variants (Supplementary Fig. S6).

Our targeted region is composed of both exonic and inter or intra genic regions. For 1669 variants in exonic regions we were able to run the above comparison with ExAC^16^ database, which is much larger (60,706 unrelated individuals) and hence more robust. The concordance is again excellent between poolAF and ExAC AF (R^2^ = 0.970; Supplementary Fig. S7). Therefore, taking advantage of publicly available variant databases reporting allele frequency, we are able to demonstrate the close similarity between our data and those collected from populations of similar or larger size.

### Validation of Pool-seq AF: comparison with Immunochip SNP-array

**Figure 3:**
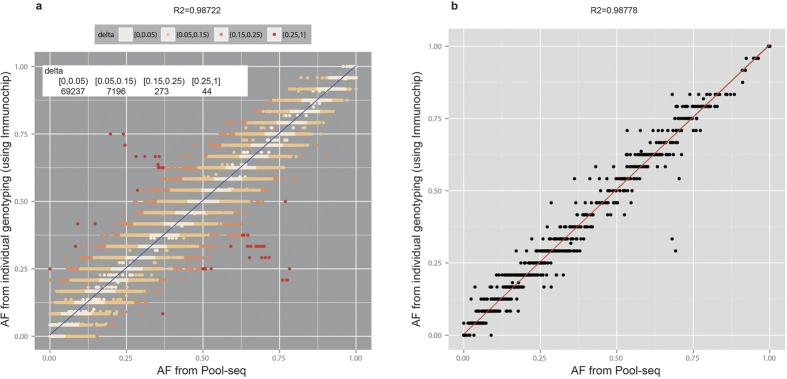
Pool sequencing AF vs. AF obtained from individual genotyping by ImmunoChip SNP-array. **(a)** Correlation scatterplot. The points are colour coded according to the absolute difference (delta) between the two frequencies; the number of points for corresponding ranges of delta is shown in top left inset. **(b)** Pool-by-pool correlation. A representative scatter plot for one of the pools (12 individuals) for 1535 SNVs is shown.

The subjects of 50 pools (out of total 83) for a total of 600 individuals have been each genotyped *individually* using Illumina’s Immunochip^20^,^21^,^22^ SNP-genotyping platform. The Immunochip platform tested 1535 variants covered in our targeted sequencing, for which a comparison was possible between the two platforms. AFs obtained from Pool-seq show an excellent correlation (R^2^ = 0.987) with AFs obtained from individual genotyping, with majority of the AF-pairs (N = 69237, 90.32%) differing by <0.05 (~1 varied chromosome out of total 24 autosomes) between two sets [Fig. 3(a)]. The relative differences (absolute delta/AF) are also small both for common as well as rare variants (Supplementary Fig. S8). In addition, the pool-by-pool correlation was very high: the mean correlation for all pools was 0.987 ± 0.001 [Fig. 3(b)]. These results further show that the estimation of AF in Pool-seq is reliable and robust.

### Sequencing errors and rare alleles

Although the previous observations suggest an overall accuracy in variant calls of known variants owing to the availability of reference public datasets and Immunochip SNP-array data of individual subjects, the same cannot be stated for “novel” rare variants that represent the vast majority of our calls. False rare variants are the most challenging problem of Pool-seq, as sequencing errors confound with alleles present at low frequencies, possibly generating many false positives. NGS technologies are not completely error-free with sequencers showing varying degree of error rates^10^,^11^. In individual sequencing, it is easy to correct for small sequencing errors as the alternative allele can take only a few discrete values (e.g. the AF of an allele in a diploid organism can only be: 0 = not present; 0.5 = heterozygous; or 1 = homozygous). However, if n diploid organisms are pooled for sequencing, the possible AFs can take any value from the set {0, 1, 2, ….., 2n − 1, 2n}/(2n). For large pool-size AF can take many possible values making it difficult to correct for small deviations in AF due to sequencing errors. Consequently, this could strongly affect AF in the low frequency range making it difficult to discriminate “real” rare variants from background noise caused by sequencing errors. Therefore, proper filtering approach has to be devised in order to remove false positive variants.

### Filtering guideline using Kolmogorov-Smirnov (KS) test: Quality Filter (QF)

**Figure 4:**
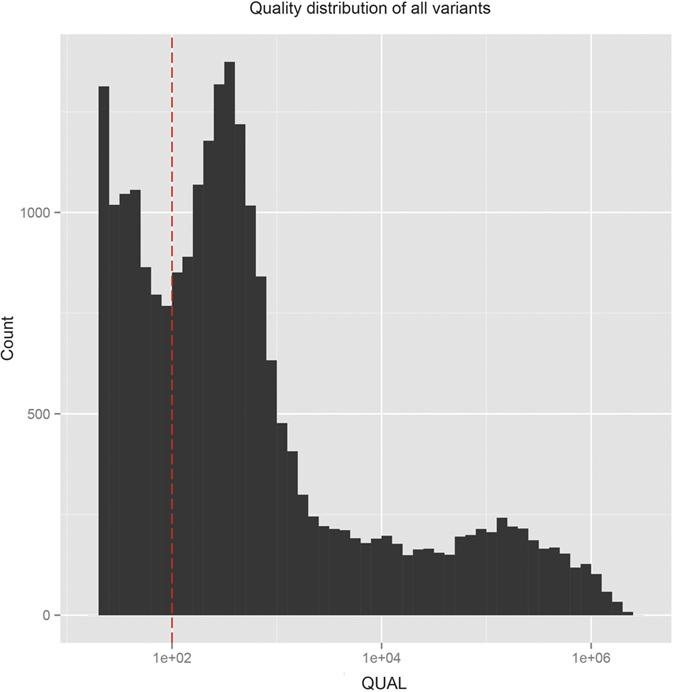
QUAL(ity) score distribution of all variants. The dashed red vertical line denotes the ad-hoc threshold of low-quality (QUAL = 100).

**Figure 5:**
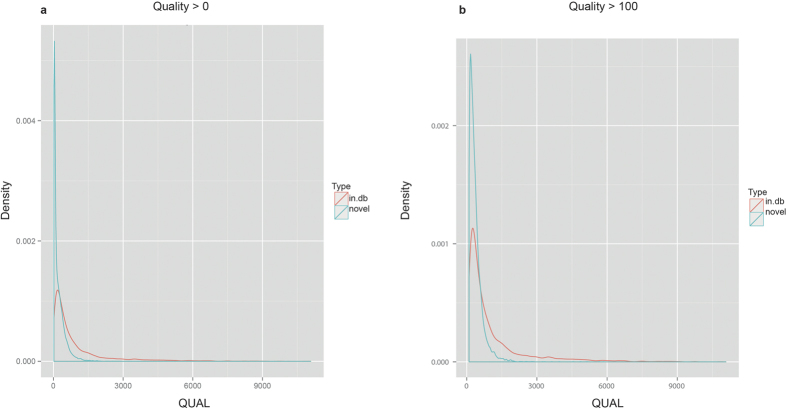
Density distributions of QUAL(ity) scores of variants found in public databases (in.db), and those not found in any database (novel). (**a**) Distributions for all variants (QUAL > 0) (**b**) Distribution for variants having QUAL > 100.

CRISP generates a quality score for each variant by considering several parameters using a sophisticated multi-step algorithm^12^. Considering our entire SNV dataset, the resulting quality score (QUAL) values span a large range, from 20 to over 1 million, distributed as shown in Fig. 4. Around 29% (N = 6862) of the variants have a “low” (QUAL < 100) quality score (Fig. 4) and almost all of them are rare variants (AF < 0.01; Supplementary Fig. S9). However, not all rare variants (N = 19139) have low quality values, actually spanning from 20 to 11080 (Supplementary Fig. S10). Comparing the distribution of quality for the rare variants reported in any of the 1000genomes, dbSNP, ExAC or ESP database (N ***in.db*** = 6359) with those not annotated in any public database (N ***novel*** = 12780), we found a disproportionate number of lower quality variants in the novel rare variant category [Fig. 5(a)]. However, we expect these two distributions to be similar because the presence or absence of variants in public database and the quality score of variant calls are completely independent parameters.

The above considerations would suggest to apply a quality based filtering, which is a very common way to remove false positive calls^23^,^24^. Indeed, applying an ad-hoc QUALITY filter of 100 (i.e. QUAL > 100) would make those distributions qualitatively more similar [Fig. 5(b)], at the cost of losing 11.92% (N = 758) of the annotated variants and 47.97% (N = 6130) of the novel ones. Assuming that annotated variants are somewhat “real” as they have been found in other databases, we would not like to lose a lot of them. So, in order to set the quality cutoff value in an objective manner, we used Kolmogorov-Smirnov (KS) test, which measures the similarity between two distributions. The D-statistics (Dstat, 0 ≤ Dstat ≤ 1) of KS-test gives a quantitative measure of the similarity between the two distributions being compared; lower values indicate more similar distributions. We ran KS-tests for “in.db” and “novel” categories at different quality cut-off thresholds, from 20 to 200 in steps of 1, and found that the Dstat is minimum (Dstat_min_ = 0.3114) for QUAL threshold of 74 (Supplementary Fig. S11). Only 7.69% (N = 489) of the annotated variants, but 42% (N = 5368) of the novel variants are removed at this Quality Filter.

### Minimum Percentage of reads Filter (MPF) as an alternative filter

Our pools are composed of 12 individuals (24 autosomes), therefore, the “ideal” lower detection limit for variant alleles in individual pools is 1/24 (AF = ~0.04). Considering that CRISP calls a variant with minimum of 4 variant reads, at high coverage a variant could be called by CRISP even if the number of variant reads is well below the theoretical threshold of 4%, possibly generating spurious variants resulting from sequencing noise. To remove these kinds of false variants due to possible sequencing errors, we recalculated the AFs in individual pools by introducing a threshold on the minimum % of reads of alternative alleles, from 0% to 5% in steps of 0.1% (see methods). We then applied the KS-test for “in.db” and “novel” categories as before at different MPF cut-off thresholds, and found that for threshold = 2.6%, the Dstat is minimum (Dstat_min_ = 0.3062) (Supplementary Fig. S12). Only 4.96% (N = 316) of the annotated variants, but 38.74% (N = 4951) of the novel variants are removed at this minimum percentage filter (MPF).

### Getting the best out of two filters

The Quality Filter (QF) and the Minimum Percentage Filter (MPF) act at different levels: QF removes a variant altogether from all of the pools if its quality is below threshold whereas MPF removes variants from only those pools where variant allele read count is below a minimum percentage of total reads. However, they do similar jobs of removing spurious variants, which is clear from the fact that using either of them makes the two distributions more similar as evidenced by lower Dstat values of KS-tests. In fact, it is striking that by using either of them, the minimum Dstat achieved is similar: 0.3062 for MPF vs. 0.3114 for QF. Also, the remaining variants after applying either filter overlap substantially (N_MPF = 13873; N_QF = 13283, N_common = 12311). This suggests that a robust way to select the bona-fide variants is to take those that are present in both of them (intersection of the two sets). This gives a Dstat of 0.309 for KS-test, which is close to the minimum Dstat of either of the two filters QF or MPF.

### Validation of filters

To ascertain the accuracy and efficacy of our filters, we *individually* sequenced all the subjects of one of the pools that contained relatively high number of rare variants before filtering. We then called the variants in all these individual samples constituting the pool using the standard GATK^25^ caller and compared them with the variants called by Pool-seq. Assuming individual variants as true, the unfiltered Pool-seq data shows a high rate of false positive (FPR = 53.97%, Table 1). Applying both MPF and QF filters on all the variants gives a reasonable false positive rate (FPR) of 6.67%, while the percentage of true positive variants retained are 96.39%. The efficacy of filters is better for common variants than rare variants (Table 1). Nevertheless, the filters are able to remove the vast majority of false variants in either case while retaining majority of true positive variants.

### Possible applications of filtering guideline

The guideline proposed here to determine cut-off values of filters is fairly generic and easy to apply. Essentially, it requires comparing the distributions of Qualities (or possibly other suitable discriminant) of “in.db” and “novel” class of variants for various filters, which can be done by any standard statistical package. We have used the function “*ks.test*()” implemented in R^26^ for it. In our case, we found that both QF and MPF are needed to clean the data. However, in other situations and depending on a different variant caller or different composition of pools, it might be possible that just one filter is sufficient enough, or a completely new combination of two or more filters is required. The important point we would like to emphasize here is that using this method gives us the opportunity to determine the cut-off values of filters objectively, which is surely a frustrating task. Moreover, this idea can be conceptually applied to even individual sequencing when a high number of false positive variants are suspected, as this method or guideline does not really depend on the fact that the variants are called in pools.

## Conclusions

Pool-seq can be successfully used as a cost-effective alternative to individual sequencing for population genetics studies. We have shown that the estimation of AF in Pool-seq is robust and reliable even with a modest pool-size of 12^6^,^7^. Sequencing noises might give rise to many spurious rare variants in Pool-seq and proper care should be taken to remove them before doing any kind of association studies involving rare variants. Our proposed filtering guideline using known variants as a reference in order to filter false positive variants is effective in removing spurious variants. This method could be adopted in similar studies of Pool-seq or even in individual sequencing to filter false positive variants.

## Methods

### Pooling and sequencing of target-regions

The target regions (total 1.9 Mb) are either whole genomic segments (17 regions) or only the coding part of genes (72 genes). DNA was collected from voluntary Italian subjects (41% male, 59% female), excluding any of Sardinian origin, and was pooled as 12 individuals/pool in 84 pools. The target regions were captured using Agilent SureSelect target enrichment method. The DNA quantity has been properly balanced in each pool in order to equally represent each genome. After the fragmentation of each DNA pool using the Covaris shearing system (Covaris inc., Massachusetts, USA), the ligation to specific paired-end adaptors, the preparation of amplified libraries and their hybridization to capture probes were performed. The enriched libraries were then subjected to Illumina protocols for cluster generation and massive sequencing. Paired-end multiplexed sequencing was performed on the Illumina GaIIx platform (Illumina, San Diego, CA), combining 6 pools tagged with different index sequences in each lane and producing 2 × 85 bp read lengths. At QC step, some samples were found to be not covered and/or sequenced properly and they were repeated for sequencing.

This study was approved by the ethics committees of San Raffaele Hospital (Milan, Italy) and AOU Maggiore della Carità (Novara, Italy). Sample and clinical information were collected after obtaining a written informed consent from all subjects in accordance with the approved guidelines. The experiments and other methods were carried out in accordance with the approved guidelines.

### Bioinformatic pipeline

Supplementary Fig. S13 shows a schematic overview of the bioinformatic pipeline. Briefly, the raw-reads are first checked for QC using FastQC^27^. The QC-checked paired end (PE) reads of each pool were mapped to NCBI human reference genome (build GRCh37) using BWA^28^ (v0.7.5a-r405). The mapping was done allowing for maximum 3 mismatches and with other default parameters of BWA. Using *samtools*^29^, we then remove the duplicate reads due to PCR amplification during library preparation. For each pool, we retain only high quality (HQ) alignments in sorted BAM files (HQ-BAM) by filtering out unmapped reads and those alignments with mapping quality (MAPQ) less than 15. These high quality alignments (HQ-BAMs) are then checked for overall mapping statistics (mapping-QC) by an in-house script. The detailed mapping statistics for each pool is reported in Supplementary Figs S2 and S3. There were three cases where the mapping-QC report suggested a very low mean depth or coverage of target regions. For those cases, the sequencing experiment was repeated. One of the pools could not pass the QC even after repeated sequencing and consequently was removed from any further analysis. All the HQ-BAMs passing the QC are ready now for variant calling.

### Variant call using CRISP and variant annotations

We have used CRISP^12^ (v27122013) as the variant caller. CRISP takes as input the BAM files (HQ-BAMs in our case) of individual pools and the reference human genome in standard FASTA format. To call the variants only in the targeted regions, it also requires the coordinate of targeted regions in UCSC BED-file format^30^. CRISP was run with all the default parameters, which are somewhat less stringent. This was deliberately done so as not to loose any potential variant due to the stringent parameters.

CRISP reports the variants in a VCF-file. We have used ANNOVAR^31^ to annotate the variants. 1000g2014sep version of Annovar table was used for 1000Genomes AFs, which is based on the data of phase III (2013.05) alignment. The Annovar table versions for other AF annotations are as follows – dbSNP: avsnp142 (v.2014.12.28); ESP: esp6500siv2 (v.2014.12.22); and ExAC: exac03 (v.2015.11.29).

### Comparison with Immunochip SNP-array

The subjects of 51 pools (out of total 83) had been each genotyped individually using Illumina’s Immunochip SNP-genotyping platform^20^,^21^,^22^. There were 1535 genomic positions common in targeted sequencing and Immunochip, for which a comparison was made between two platforms. The pool-by-pool correlation plot suggested that one sample (pool) had a low correlation (R^2^ = 0.872) compared to all other pools (mean R^2^ = 0.987 ± 0.001), for which it was dropped from the overall correlation analysis. The coefficient of determination R^2^, which is numerically equal to the square of Pearson correlation coefficient, was obtained using *lm*() function of R^26^ and plotting was done using ggplot2 library of R. The R^2^ for all the 51 pools have been reported in Supplementary Table S2.

### AF calculation and AF with MPF filter

The allele frequencies have been calculated as the average AF of all the pools. The AF in individual pools has been calculated as the fraction of total number of reads supporting the alternative allele.

The AFs using minimum percentage filter (MPF) have been calculated like this: for any pool having alternative allele (ALT) reads less than *threshold* = *th%* value, its ALT reads value has been reset to 0 (assuming it as sequencing error). We then re-calculate the AFs of variants for different values of thresholds from the set th = {0.1, 0.2, 0.3, …., 4.7, 4.8, 4.9, 5.0}%.

### Kolmogorov-Smirnov (KS) test

The KS-test was used to compare the distributions of quality scores of two classes of variants, namely, variants present in any public database (in.db) and “novel” variants. The advantage of using KS-test is that it is a non-parametric test; hence, it does not require or assume any information about the types of distributions compared. The function *ks.test*() from standard implementation of R^26^ was used for it. The D-statistics (0 ≤ Dstat ≤ 1) of KS-test gives a quantitative measure of the differences between the two distributions; lower values indicate more similar distributions.

### Comparison with individual sequencing variants of one of the pools

The subjects of one of the pools were each sequenced individually using exactly the same procedure of capture and sequencing as followed for the pools. The pool for individual sequencing was selected based on a very high number of rare variants and a very good mean depth. The variants in the individual samples of that pool were called *together* using GATK^25^ (using HaplotypeCaller in GVCF mode), following the developers’ recommendations and default parameters. We then compared these variants with CRISP variants in that pool. The various comparison parameters are calculated as follows:


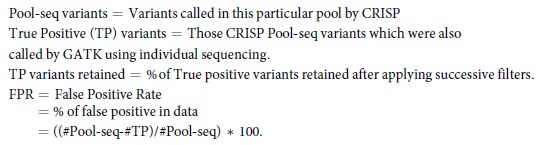


### Analysis tools

The analyses were done using statistical programming language R^26^ and custom Unix shell scripts. A series of custom R-scripts were written to do the comparison of AF with public databases and with Immunochip, to find the correlation of AF with Immunochip, to find the AF after applying QF and MPF, to run the KS-tests etc. The scripts are available upon request. Plotting was done using *ggplot2* library of R.

The mean values are shown as mean ± standard error of mean (s.e.m.), unless specified otherwise.

## Additional Information

**How to cite this article**: Anand, S. *et al*. Next Generation Sequencing of Pooled Samples: Guideline for Variants’ Filtering. *Sci. Rep*. **6**, 33735; doi: 10.1038/srep33735 (2016).

## Supplementary Material

Supplementary Information

## Figures and Tables

**Table 1:** Summary of comparison of Pool-seq variants with variants obtained from individual sequencing of the same pool (before and after filtering).

Variant Type	Filters Applied	Pool-seq variants	TP variants	TP variants retained	FPR
*ALL Variants*	Original Variants	8195	3772	100.00	53.97
	After MPF & QF Filters	3896	3636	96.39	6.67
*Common Variants*	Original Variants	3911	3406	100.00	12.91
	After MPF & QF Filters	3566	3326	97.65	6.73
*Rare Variants*	Original Variants	4284	366	100.00	91.46
	Both MPF and QF	330	310	84.70	6.06

Original Variants = Original number of variants without any filter. MPF = Minimum Percentage Filter; QF = Quality Filter. Pool-seq variants = number of variants called by CRISP in this pool. True Positive (TP) variants = Number of pool-seq variants confirmed by individual sequencing. TP variants retained = % of TP variants retained after applying the respective filters. False Positive Rate (FPR) = Rate of False positive variants in respective data (before or after filters). See methods for details about TP and FPR calculations.
