# Author Correction: Next Generation Sequencing of Pooled Samples: Guideline for Variants’ Filtering

**DOI:** 10.1038/s41598-020-62214-5

**Published:** 2020-03-23

**Authors:** Santosh Anand, Eleonora Mangano, Nadia Barizzone, Roberta Bordoni, Melissa Sorosina, Ferdinando Clarelli, Lucia Corrado, Filippo Martinelli Boneschi, Sandra D’Alfonso, Gianluca De Bellis

**Affiliations:** 1 Institute for Biomedical Technologies, National Research Council, Segrate (MI), Italy; 2 Department of Science and Technology, University of Sannio, Benevento, Italy; 3 Interdisciplinary Research Center of Autoimmune Diseases IRCAD, University of Eastern Piedmont, Novara, Italy; 4 Department of Health Sciences, University of Eastern Piedmont, Novara, Italy; 5 Division of Neuroscience, Laboratory of Human Genetics of Neurological Disorders, Institute of Experimental Neurology (INSPE), San Raffaele Scientific Institute, Milan, Italy; 6 Department of Neurology, Division of Neuroscience, Scientific Institute San Raffaele, Milan, Italy; 7 Present Address: Department of Genetic Medicine and Development (GEDEV), University of Geneva Medical School, 1 Rue Michel-Servet, Geneva, 1211 Switzerland

Correction to: *Scientific Reports* 10.1038/srep33735, published online 27 September 2016

This Article contains an expired email address for Santosh Anand. Correspondence and requests for materials should be addressed to santosh.anand@gmail.com.

