# Supplementary material for: Next Generation Sequencing of Pooled Samples: Guideline for Variants’ Filtering
Source: Sci Rep. 2016 Sep 27;6:33735. doi: 10.1038/srep33735 (PMC5037392; doi:10.1038/srep33735)
Supplement: Supplementary Information [file srep33735-s1.pdf]

Supplementary Information

Next Generation Sequencing of Pooled Samples: Guideline for Variants' Filtering

Santosh Anand[1,2,+,*], Eleonora Mangano[1,+], Nadia Barizzone[3,4,+], Roberta Bordoni[1], Melissa Sorosina[5], Ferdinando Clarelli[5], Lucia Corrado[3,4], Filippo Martinelli Boneschi[5,6,#], Sandra D'Alfonso[3,4,#], Gianluca De Bellis[1,#]

[1]Institute for Biomedical Technologies, National Research Council, Segrate (MI), Italy.
[2]Department of Science and Technology, University of Sannio, Benevento, Italy.
[3]Interdisciplinary Research Center of Autoimmune Diseases IRCAD, University of Eastern Piedmont, Novara, Italy.
[4]Department of Health Sciences, University of Eastern Piedmont, Novara, Italy.
[5]Laboratory of Genetics of Complex Neurological Disorders, Institute of Experimental Neurology (INSPE), Division of Neuroscience, San Raffaele Scientific Institute, Milan, Italy.
[6]Department of Neurology, Division of Neuroscience, Scientific Institute San Raffaele, Milan, Italy.

*Corresponding author
E-mail: santosh.anand@itb.cnr.it (SA)

[+]These authors contributed equally to this work
[#]These senior authors also contributed equally to this work

Supplementary Figures

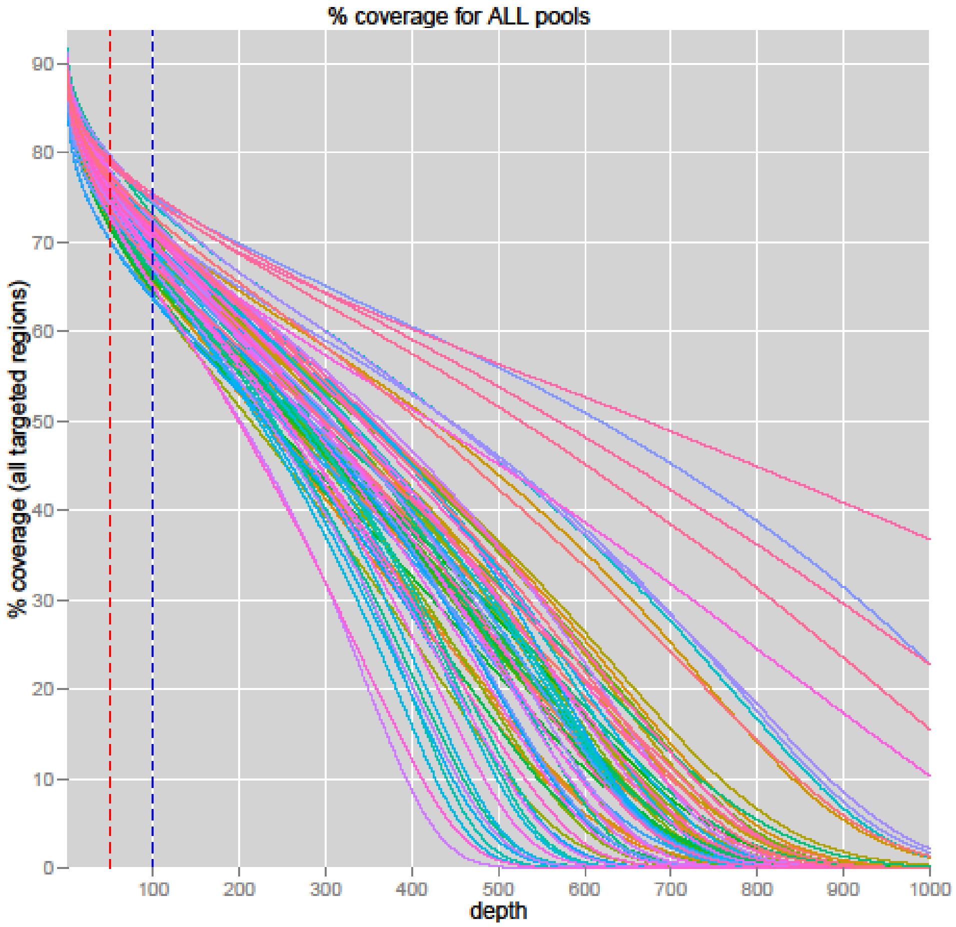

Supplementary Figure S1: Percentage coverage of all targeted regions vs. depth distribution across all the pools. Each coloured line represents a pool.

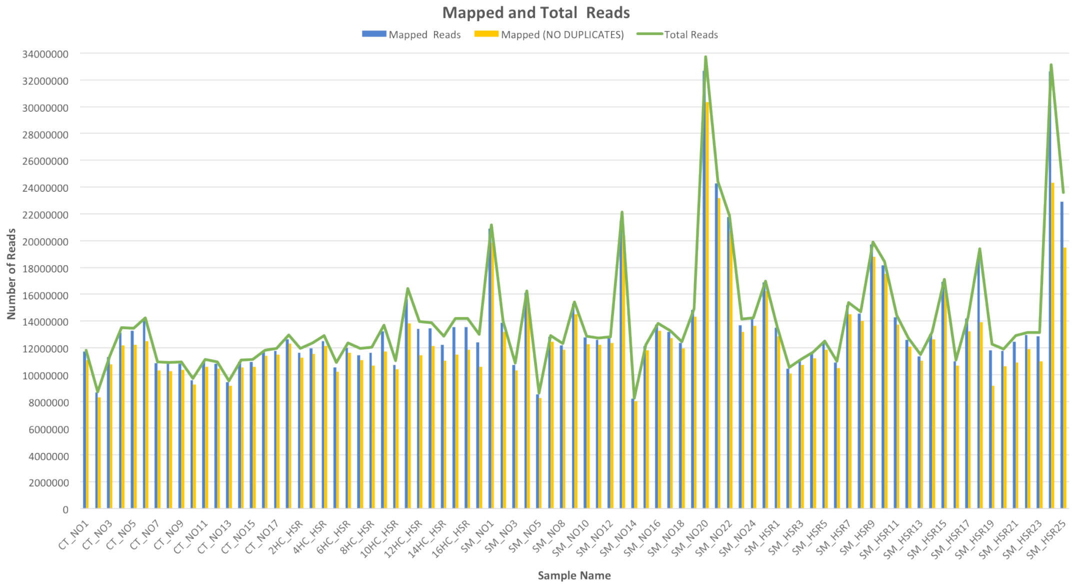

Supplementary Figure S2: Mapped and Total Reads across all pools. Mapped (NO DUPLICATES), indicated by yellow bars, are reads mapped after removal of duplicates.

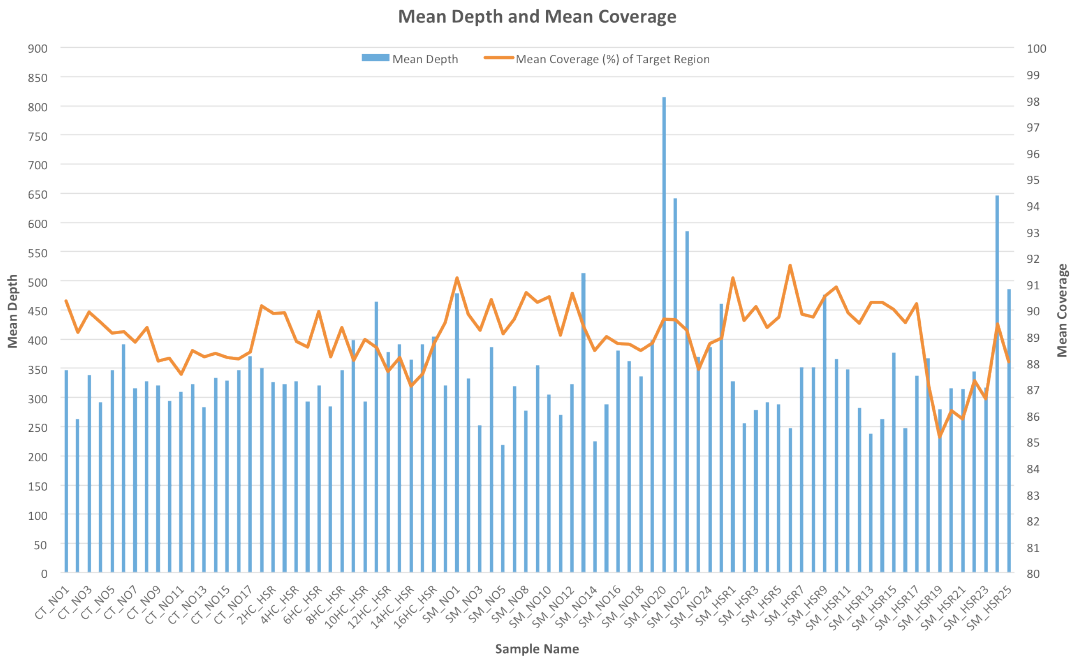

Supplementary Figure S3: Mean Depth and mean coverage (in percentage) of targeted regions across all pools.

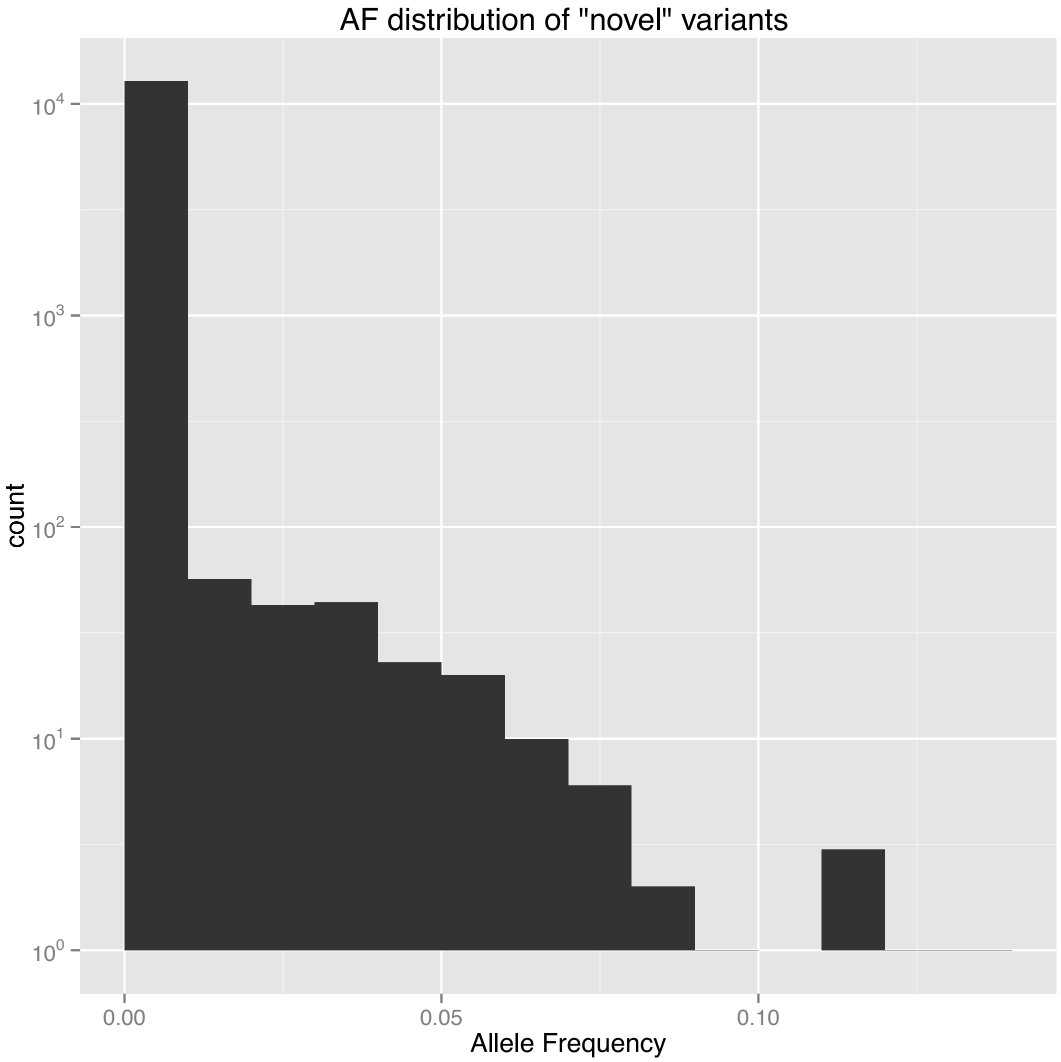

Supplementary Figure S4: Allele Frequency distribution of "novel" (variants not found in any public database) variants.

a

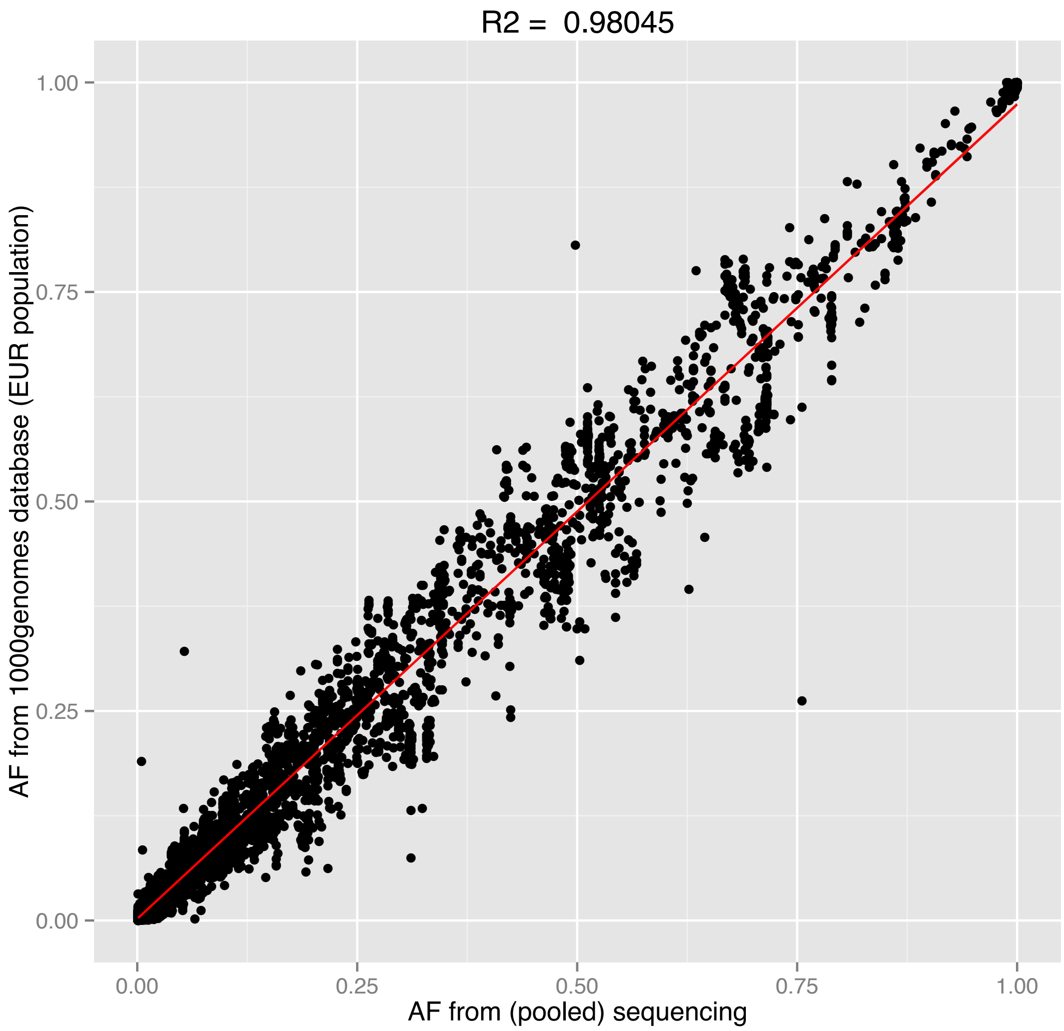
R2 = 0.98045
1.00
0.75
0.50
0.25
0.00
AF from 1000genomes database (EUR population)
0.00
0.25
0.50
0.75
1.00
AF from (pooled) sequencing

b

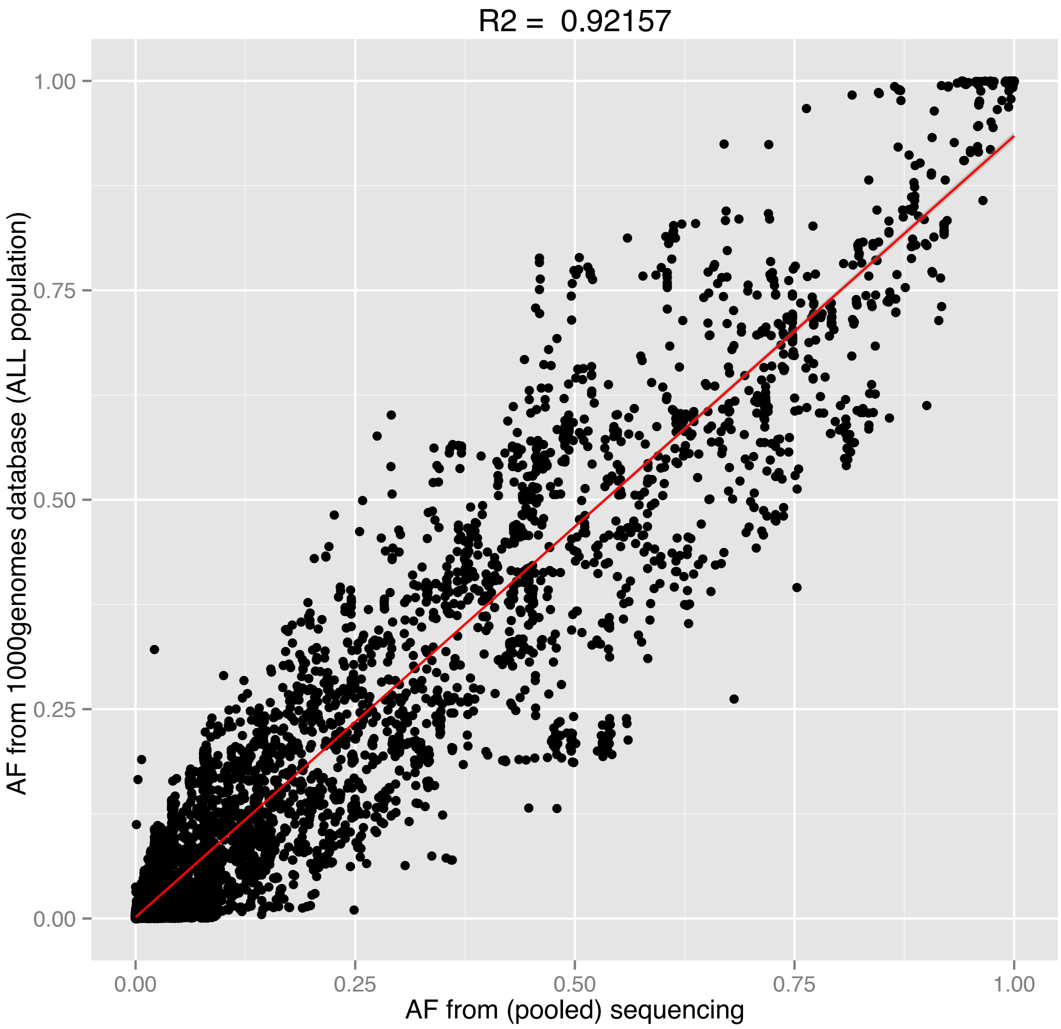

Supplementary Figure S5: Correlation scatterplot of poolAF and AF obtained from 1000genomes database. **(a)** 1000genomes_EUR **(b)** 1000genomes_ALL

a

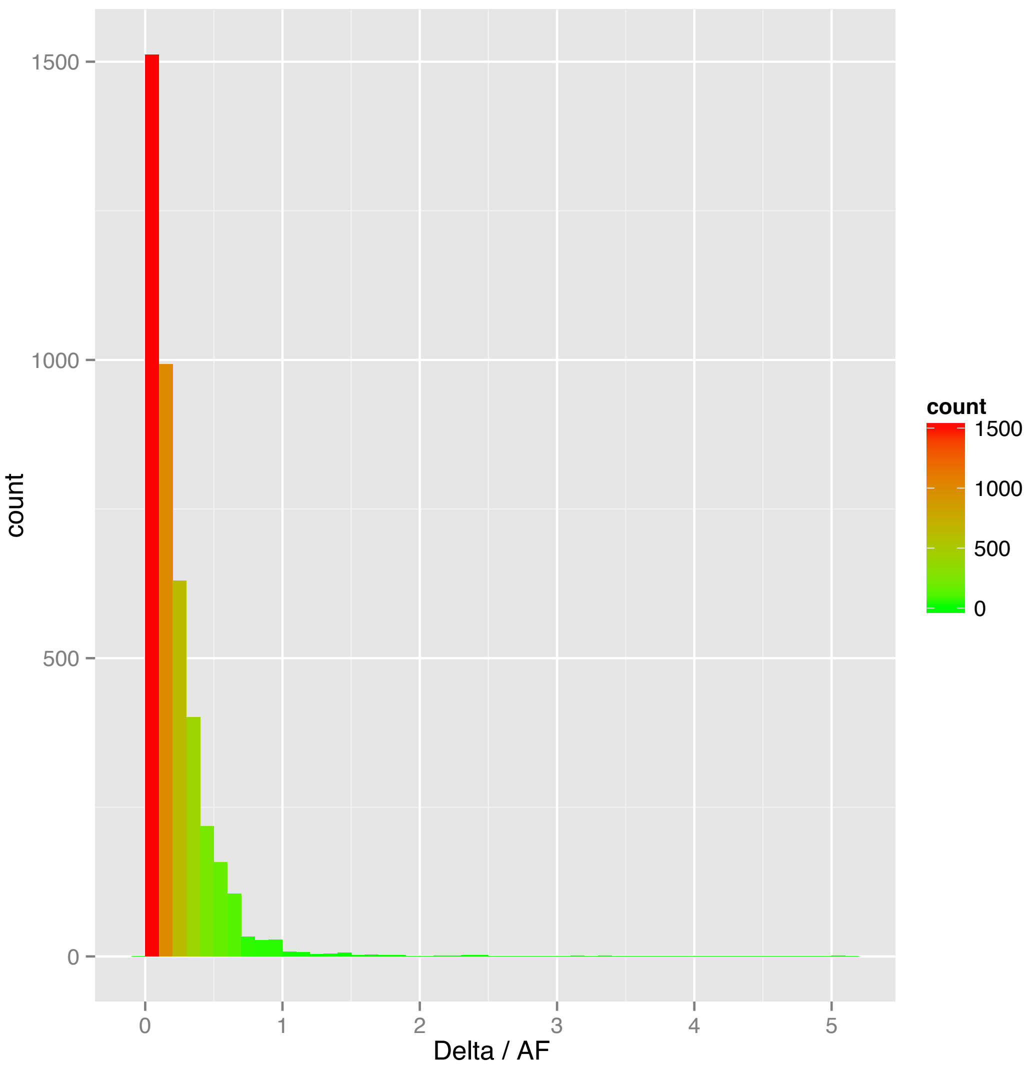

b

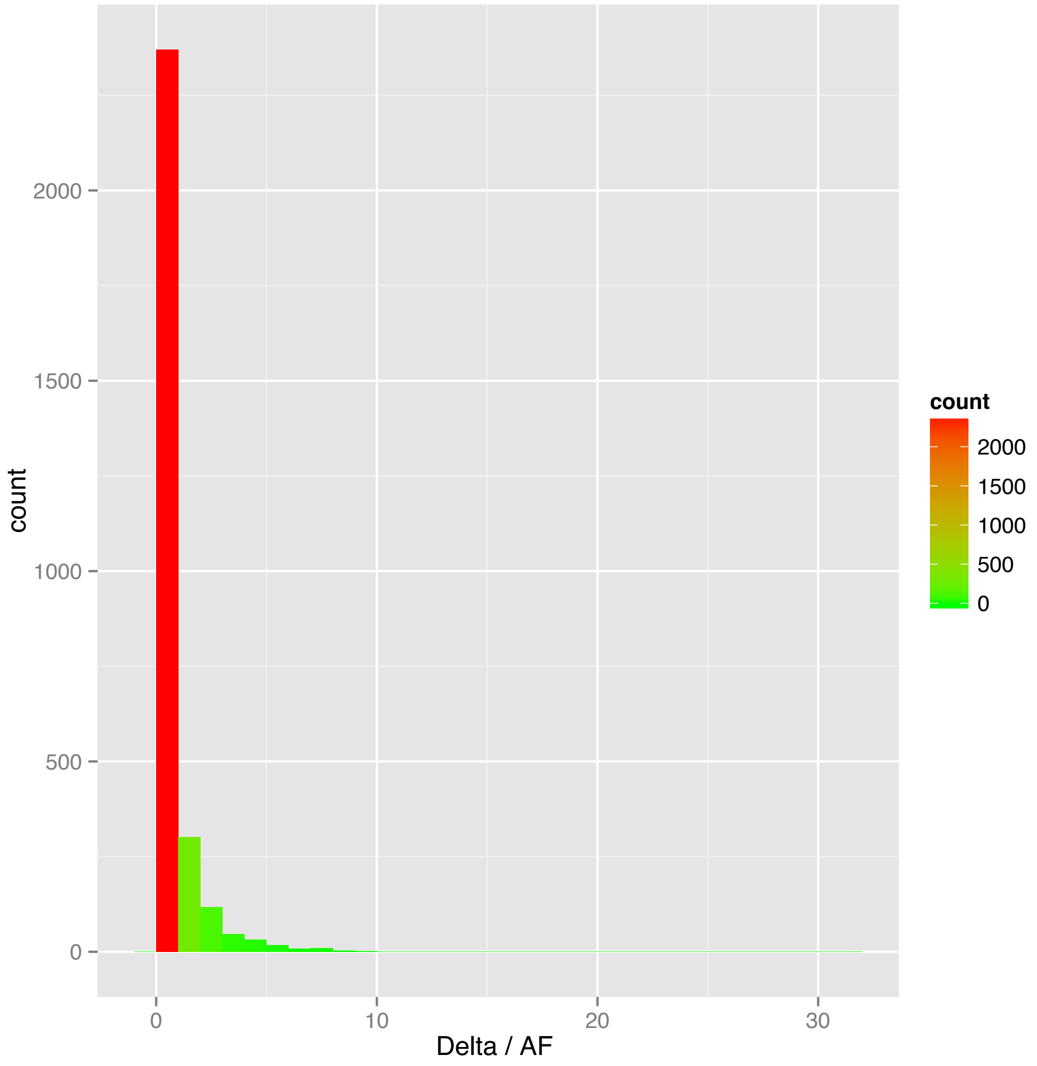

Supplementary Figure S6: Distribution of relative differences (absolute delta / AF) between poolAF and 1000genomes_EUR AF. **(a) Common variants (AF >= 0.01).** Minimum: 0.000, 1st Quartile: 0.064, Median: 0.151, Mean: 0.219, 3rd Quartile: 0.297, Maximum: 5.010. **(b) Rare variants (AF < 0.01).** Minimum: 0.000, 1st Quartile: 0.248, Median: 0.496, Mean: 0.820, 3rd Quartile: 0.797, Maximum: 30.130.

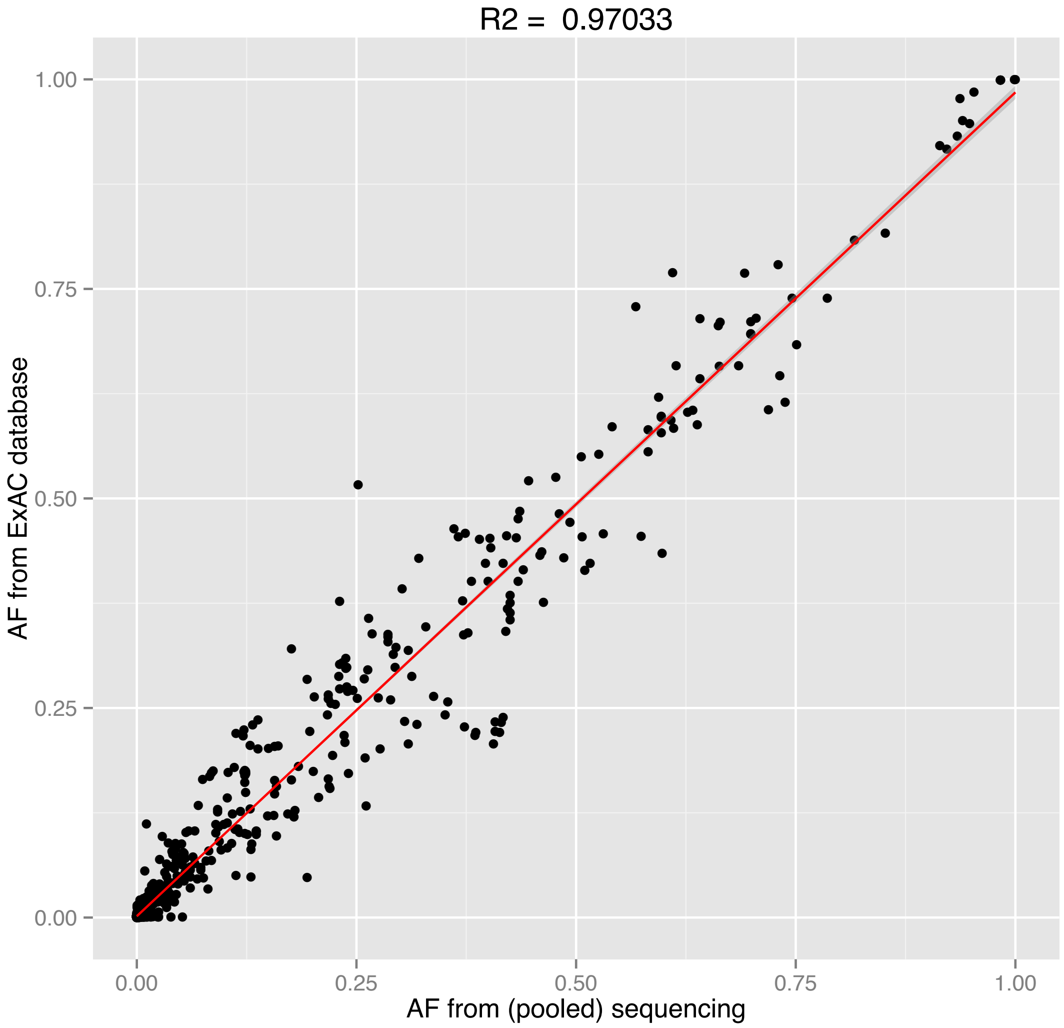

Supplementary Figure S7: Correlation scatterplot of poolAF and AF obtained from ExAC database.

a

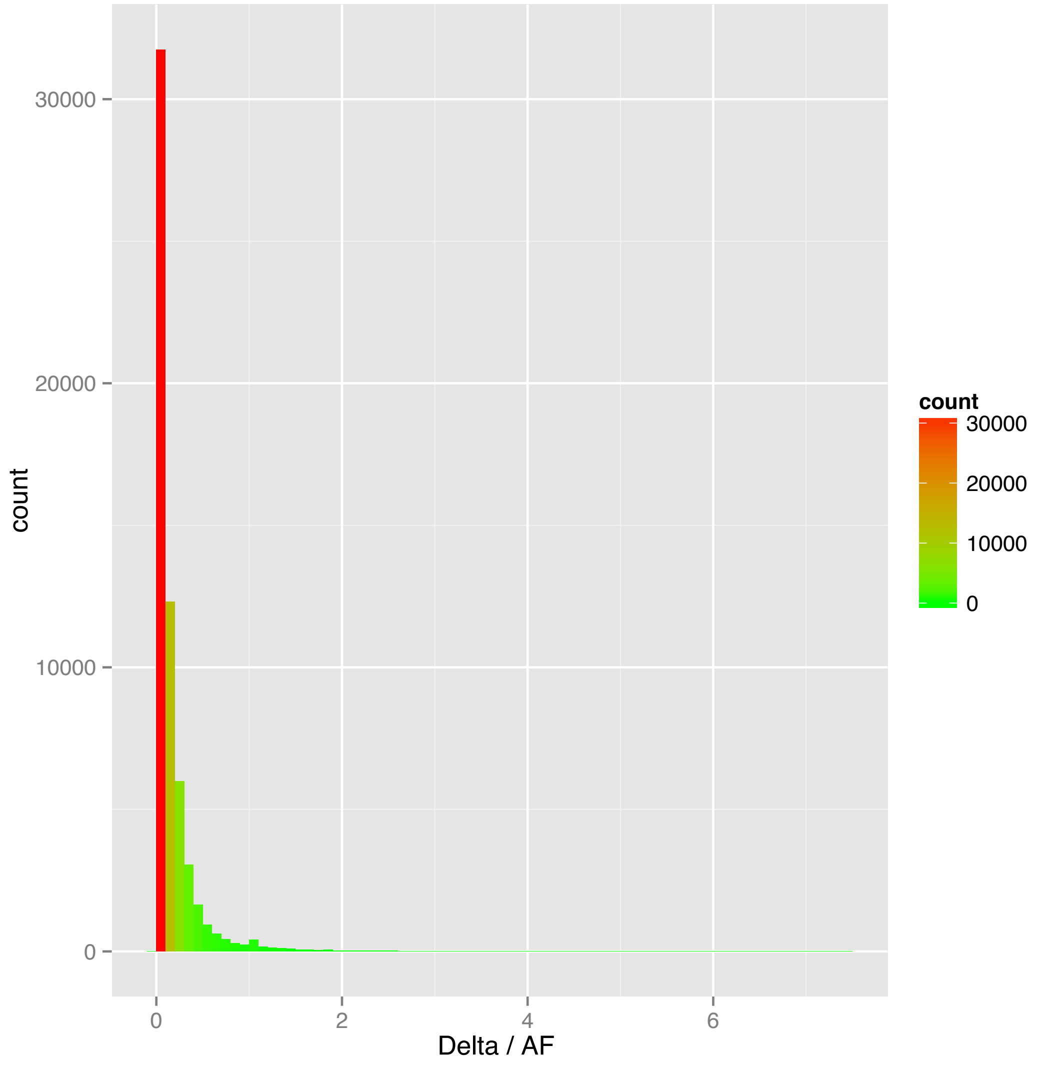
count
30000
20000
10000
0
0
2
4
6
Delta / AF
count
30000
20000
10000
0

b

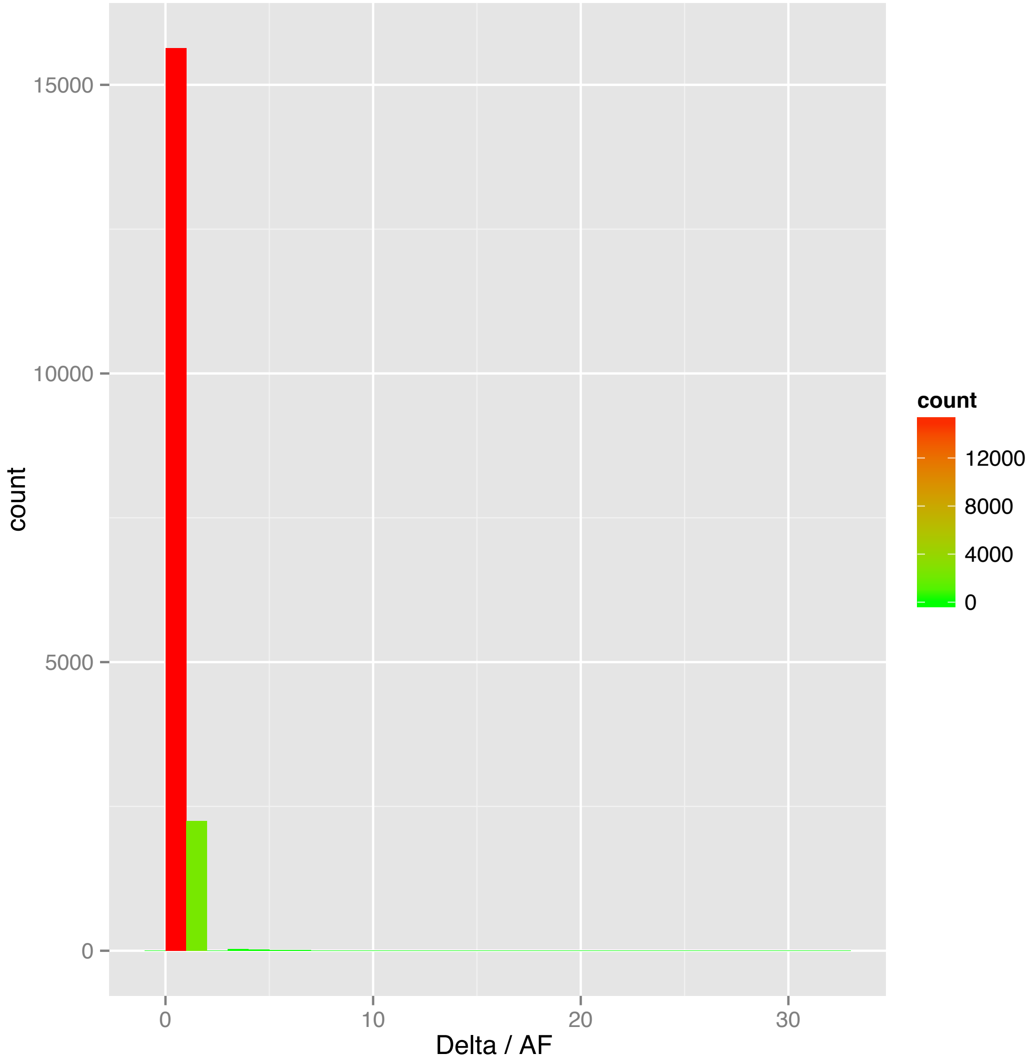

Supplementary Figure S8: Distribution of relative differences (absolute delta / AF) between poolAF and AF from individual genotyping using Immunochip. **(a) Common variants (AF >= 0.01).** Minimum: 0.000;1st Quartile: 0.031; Median: 0.087; Mean: 0.177; 3rd Quartile: 0.200; Maximum: 7.33. **(b) Rare variants (AF < 0.01).** Minimum: 0.000;1st Quartile: 0.000; Median: 0.000; Mean: 0.161; 3rd Quartile: 0.000; Maximum: 31.050.

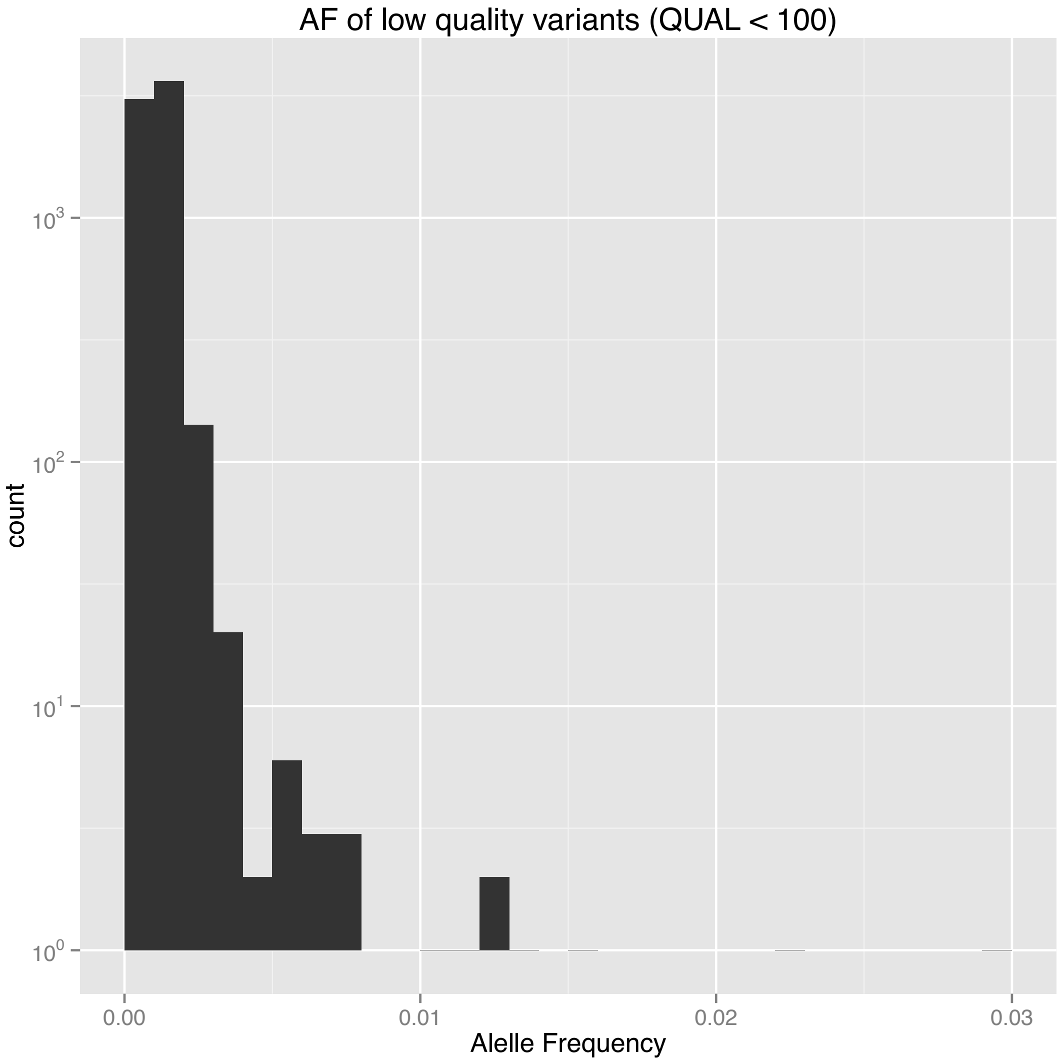

Supplementary Figure S9: AF distribution of low-quality (QUAL < 100) variants.

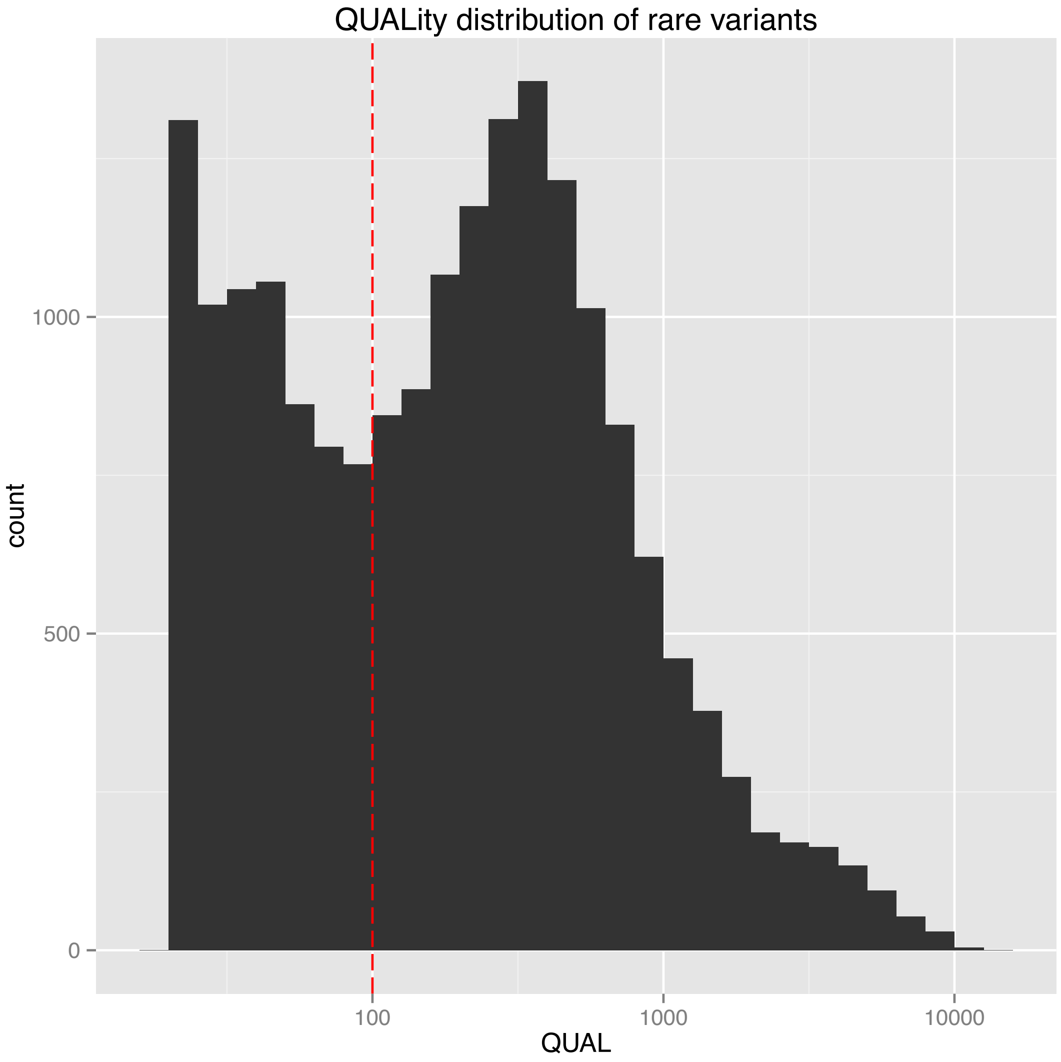

Supplementary Figure S10: QUAL(ity) score distribution of rare variants. Vertical red dashed line denotes the ad-hoc threshold (QUAL = 100) for low quality.

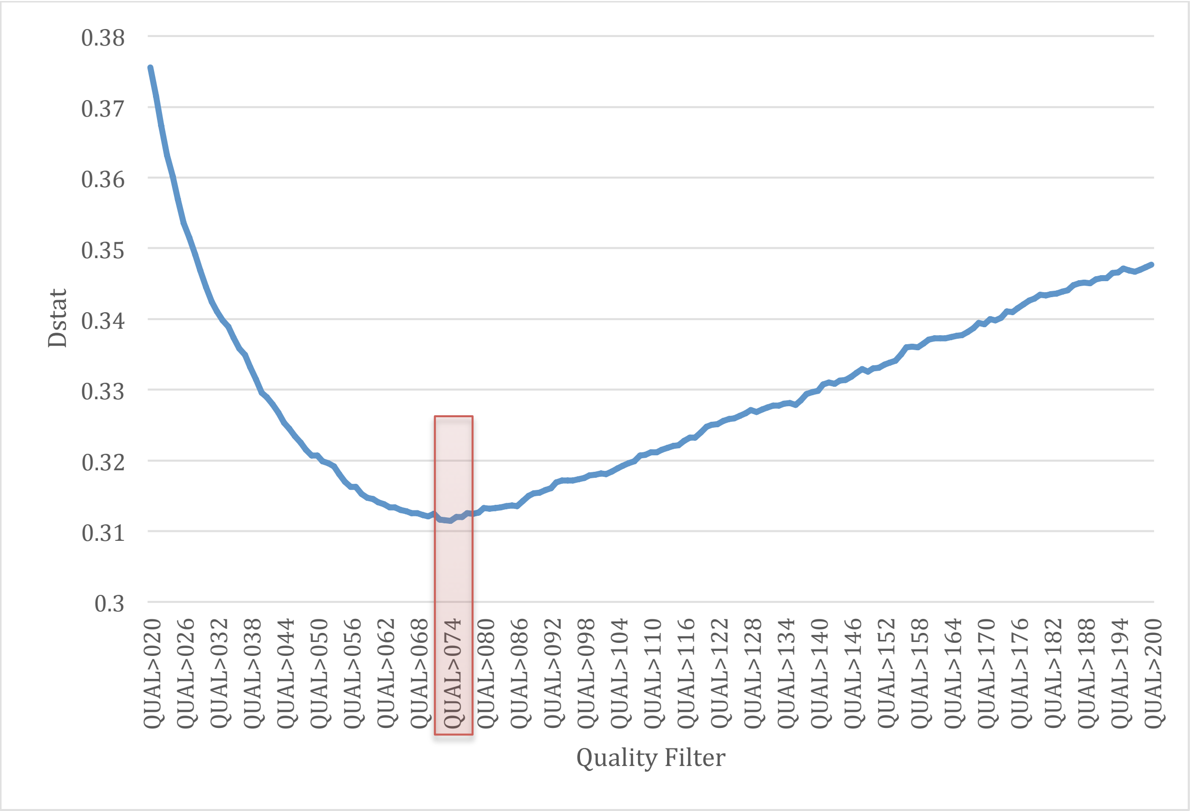

Supplementary Figure S11: KS-tests results at different Quality Filter (QF) thresholds. The minimum Dstat ($Dstat_{min}$ = 0.3114) is reached at QUAL threshold of 74 (i.e. QUAL > 74).

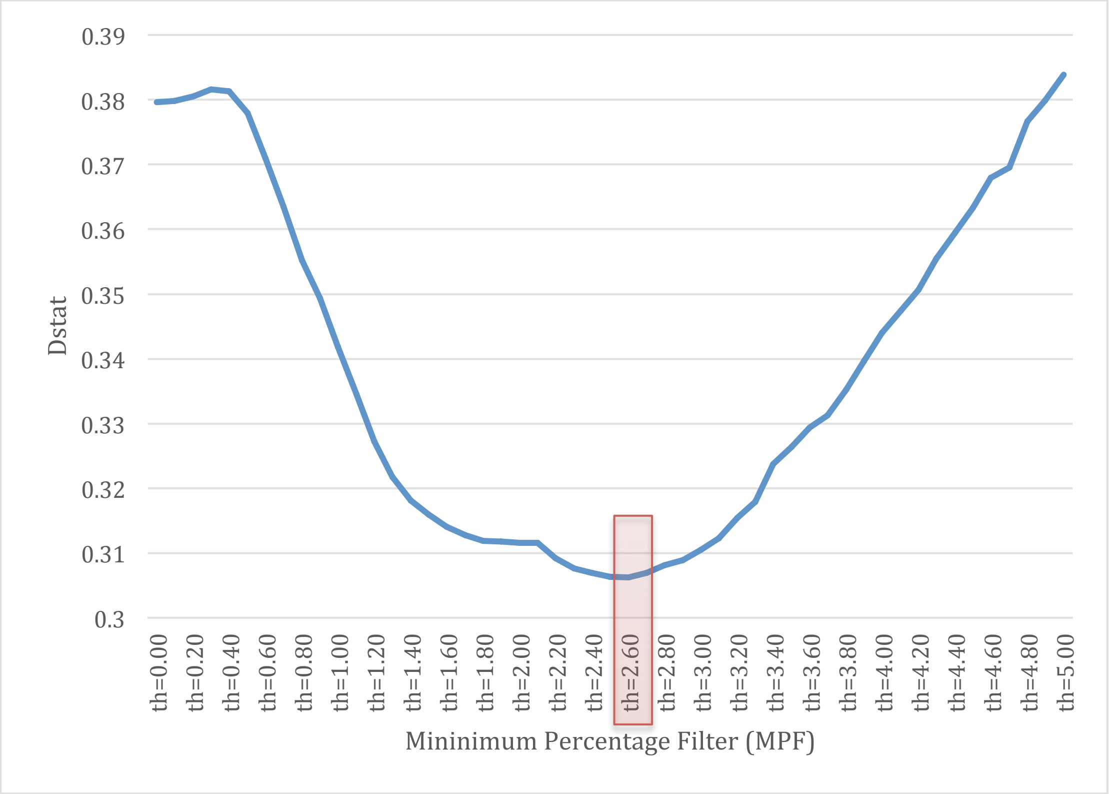

Supplementary Figure S12: KS-tests results at different thresholds of Minimum Percentage Filter (MPF) of ALT alleles. The minimum Dstat ($Dstat_{min}$ = 0.3062) is reached at MPF threshold of 2.6%.

Overview of bioinformatics pipeline

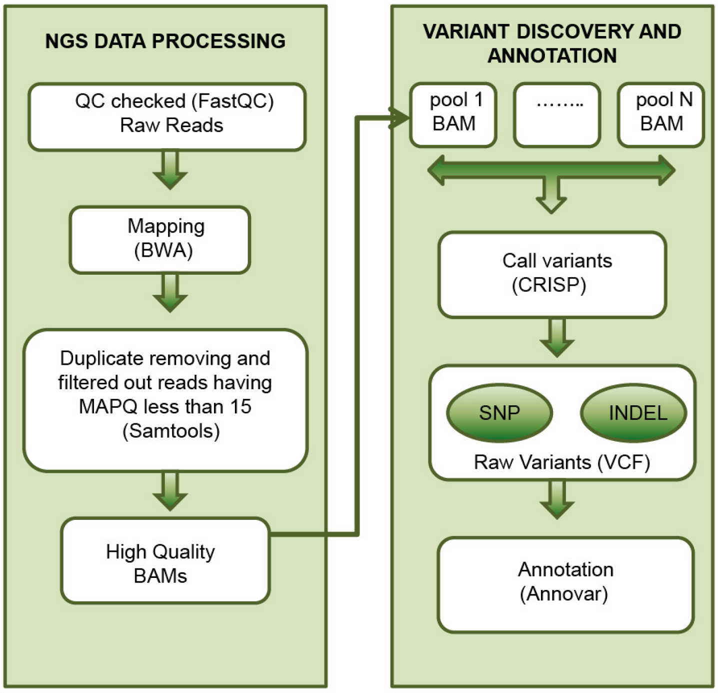

Supplementary Figure S13: Schematic overview of bioinformatic pipeline**.**

Supplementary Tables

Supplementary Table S1: Sequencing results for all pools

	Min.	1st Qu.	Median	Mean	3rd Qu.	Max.
**Total Reads**	8232918	11609659	12890954	**13963493**	14231289	33707386
**Mapped Reads**	8188029	11420748	12604589	**13718324**	14144207	32673699
**Duplicate Reads**	169082	371178	532114	**918732**	1061584	8285985
**Mapped Reads (%)**	95.16	97.27	98.89	**98.28**	99.19	99.5
**Duplicates (%)**	2.054	3.227	3.911	**5.972**	6.714	25.672
**Mapped Reads (NO DUPLICATES)**	8018947	10690320	11856557	**12799592**	13260984	30348318
**%Mapped Reads (NO DUPLICATES)**	71.72	91.55	94.6	**92.31**	95.62	97.4
**Mean Depth**	219.2	293	332.8	**351.9**	373.6	815
**Mean Coverage**	85.15	88.37	89.16	**89.06**	89.87	91.69

.

Supplementary Table S2: Pool-by-pool correlation between pooled-sequencing AF (poolAF) and AF obtained from Immunochip SNP-array.

	R^2	Remark
CT_NO1_Run58.chp|CT_NO1_Run58.seq	0.993311844	
CT_NO2_Run58.chp|CT_NO2_Run58.seq	0.985115565	
CT_NO3_Run58.chp|CT_NO3_Run58.seq	0.989815206	
CT_NO4_Run58.chp|CT_NO4_Run58.seq	0.990162551	
CT_NO5_Run58.chp|CT_NO5_Run58.seq	0.990072385	
CT_NO6_Run58.chp|CT_NO6_Run58.seq	0.992709062	
CT_NO7_Run58.chp|CT_NO7_Run58.seq	0.990531915	
CT_NO8_Run58.chp|CT_NO8_Run58.seq	0.989939293	
CT_NO9_Run58.chp|CT_NO9_Run58.seq	0.988650636	
CT_NO10_Run58.chp|CT_NO10_Run58.seq	0.989064001	
CT_NO11.chp|CT_NO11.seq	0.990991065	
CT_NO12.chp|CT_NO12.seq	0.980199122	
CT_NO13_Run58.chp|CT_NO13_Run58.seq	0.982958596	
CT_NO14.chp|CT_NO14.seq	0.986235418	
CT_NO15.chp|CT_NO15.seq	0.987778441	
CT_NO16_Run58.chp|CT_NO16_Run58.seq	0.989072283	
CT_NO17.chp|CT_NO17.seq	0.981784013	
X1HC_HSR.chp|X1HC_HSR.seq	0.987958744	
X2HC_HSR.chp|X2HC_HSR.seq	0.989730352	
X3HC_HSR.chp|X3HC_HSR.seq	0.987626275	
X4HC_HSR.chp|X4HC_HSR.seq	0.986311237	

X5HC_HSR.chp|X5HC_HSR.seq	0.987465177	
X6HC_HSR.chp|X6HC_HSR.seq	0.983855943	
X7HC_HSR.chp|X7HC_HSR.seq	0.988633208	
X8HC_HSR.chp|X8HC_HSR.seq	0.992620553	
X9HC_HSR.chp|X9HC_HSR.seq	0.990274638	
X10HC_HSR.chp|X10HC_HSR.seq	0.979022206	
X11HC_HSR.chp|X11HC_HSR.seq	0.992873813	
X12HC_HSR.chp|X12HC_HSR.seq	0.990276008	
X13HC_HSR.chp|X13HC_HSR.seq	0.992747882	
X14HC_HSR.chp|X14HC_HSR.seq	0.991196387	
X15HC_HSR.chp|X15HC_HSR.seq	0.985013499	
X16HC_HSR.chp|X16HC_HSR.seq	0.986462208	
X17HC_HSR.chp|X17HC_HSR.seq	0.990084718	
SM_HSR1.chp|SM_HSR1.seq	0.981120288	
SM_HSR15.chp|SM_HSR15.seq	0.9508251	
SM_HSR16.chp|SM_HSR16.seq	0.988179658	
SM_HSR17.chp|SM_HSR17.seq	0.871518768	REMOVED
SM_HSR18.chp|SM_HSR18.seq	0.980928817	
SM_HSR19_2.chp|SM_HSR19_2.seq	0.985448444	
SM_HSR20.chp|SM_HSR20.seq	0.981911516	
SM_HSR21_2.chp|SM_HSR21_2.seq	0.986584879	
SM_HSR22.chp|SM_HSR22.seq	0.988514799	
SM_HSR23.chp|SM_HSR23.seq	0.9911689	
SM_HSR24.chp|SM_HSR24.seq	0.993709213	
SM_HSR25.chp|SM_HSR25.seq	0.983262612	
SM_NO7.chp|SM_NO7.seq	0.99035054	
SM_NO8.chp|SM_NO8.seq	0.985746679	
SM_NO11.chp|SM_NO11.seq	0.989874649	
SM_NO24.chp|SM_NO24.seq	0.993547713	
SM_NO25.chp|SM_NO25.seq	0.994854551	
